# Use of Ceftaroline in Hospitalized Patients with and without COVID-19: A Descriptive Cross-Sectional Study

**DOI:** 10.3390/antibiotics10070763

**Published:** 2021-06-23

**Authors:** Daniele Roberto Giacobbe, Chiara Russo, Veronica Martini, Silvia Dettori, Federica Briano, Michele Mirabella, Federica Portunato, Chiara Dentone, Sara Mora, Mauro Giacomini, Marco Berruti, Matteo Bassetti

**Affiliations:** 1 Department of Health Sciences, University of Genoa, 16132 Genoa, Italy; chiara.russo16@icloud.com (C.R.); silvidetto@gmail.com (S.D.); federica.briano91@gmail.com (F.B.); marco.berruti1@gmail.com (M.B.); matteo.bassetti@unige.it (M.B.); 2 Clinica Malattie Infettive, San Martino Policlinico Hospital—IRCCS, 16132 Genoa, Italy; Michelemirabella90@gmail.com (M.M.); fedu90@gmail.com (F.P.); chiara.dentone@hsanmartino.it (C.D.); 3 School of Medicine, University of Genoa, 16132 Genoa, Italy; martini.vero@libero.it; 4 Department of Informatics Bioengineering, Robotics, and Systems Engineering (DIBRIS), University of Genoa, 16145 Genoa, Italy; sara.mora.1994@gmail.com (S.M.); Mauro.Giacomini@dibris.unige.it (M.G.)

**Keywords:** ceftaroline, MRSA, *Staphylococcus aureus*, COVID-19

## Abstract

A single-center cross-sectional study was conducted to describe the use of ceftaroline in a large teaching hospital in Northern Italy, during a period also including the first months of the coronavirus disease 2019 (COVID-19) pandemic. The primary objective was to describe the use of ceftaroline in terms of indications and characteristics of patients. A secondary objective was to describe the rate of favorable clinical response in patients with bloodstream infections (BSI) due to methicillin-resistant *Staphylococcus aureus* (MRSA-BSI) receiving ceftaroline. Overall, 200 patients were included in the study. Most of them had COVID-19 (83%, 165/200) and were hospitalized in medical wards (78%, 155/200). Included patients with COVID-19 pneumonia were given empirical ceftaroline in the suspicion of bacterial co-infection or superinfection. Among patients with MRSA-BSI, ceftaroline was used as a first-line therapy and salvage therapy in 25% (3/12) and 75% (9/12) of cases, respectively, and as a monotherapy or in combination with daptomycin in 58% (7/12) and 42% (5/12) of patients, respectively. A favorable response was registered in 67% (8/12) of patients. Improving etiological diagnosis of bacterial infections is essential to optimize the use of ceftaroline in COVID-19 patients. The use of ceftaroline for MRSA-BSI, either as a monotherapy or in combination with other anti-MRSA agents, showed promising rates of favorable response.

## 1. Background

Ceftaroline fosamil is approved for the treatment of complicated skin and soft tissue infections and community-acquired pneumonia [1].

The off-label use of ceftaroline has also been reported, mostly for unmet clinical needs, for example salvage therapy of persistent methicillin-resistant *Staphylococcus aureus* (MRSA) bacteremia, or for taking advantage of possible synergistic effects when administered in combination with vancomycin or daptomycin [2,3,4,5,6,7]. However, a clear picture of how ceftaroline is used in real life (e.g., proportion of patients receiving ceftaroline for on-label vs. off-label indications, empirical vs. targeted therapy, monotherapy vs. combination) is still unavailable, as is information regarding its possible use for the treatment of invasive bacterial superinfections in patients with acute respiratory failure due to coronavirus disease 2019 (COVID-19). Such a descriptive picture may help to identify the precise current real-life use of ceftaroline to guide and focus the design of dedicated randomized clinical trials. In turn, this would improve the quality of evidence on the best possible place in therapy of ceftaroline for currently off-label indications, ultimately further optimizing its use.

For this reason, we conducted a single-center, cross-sectional study to describe the use of ceftaroline in a large teaching hospital in Northern Italy, which also covered the first months of the COVID-19 pandemic.

## 2. Methods

This cross-sectional, observational study was conducted at Ospedale Policlinico San Martino IRCSS, a 1200-bed hospital in Genoa, Northern Italy. From July 2019 to December 2020, two-hundred patients receiving ceftaroline for any indication were included in the study. Ceftaroline was administered by physician according to their judgment and independent of the study protocol, in line with the observational nature of the study. All patients without COVID-19 receiving ceftaroline were enrolled consecutively; however this was ultimately not possible for COVID-19 patients during the peak of the pandemic, so patients receiving ceftaroline were enrolled whenever possible up to reaching the predefined sample size of 200 patients (see sample size calculation below).

The primary objective was to describe the use of ceftaroline in terms of indications and characteristics of patients. A secondary objective was to describe the rate of clinical response at the end of ceftaroline treatment in a longitudinal observational prospective subgroup analysis of patients with bloodstream infections (BSI) due to MRSA (MRSA-BSI).

The study was approved by the ethics committee of the Liguria Region (registry number 291/2018). According to the current privacy regulation (General Data Protection Regulation (GDPR), EU 2016/679), patients able to provide an informed consent agreed to participate in the study. A waiver of informed consent was obtained for patients unable to provide an informed consent while hospitalized (e.g., those with invasive infection who were unconscious at the time of study inclusion). The collection of anonymous descriptive clinical and laboratory data from hospitalized COVID-19 patients was also approved by the ethics committee of the Liguria Region (registry number 163/2020).

Deviations from the original study protocol consequent to the COVID-19 pandemic are listed in the Supplementary Materials.

### 2.1. Study Procedures and Definitions

In line with the cross-sectional nature of the study primary analysis, data was collected at the time of ceftaroline initiation. No follow-up was conducted, except for: (i) results of culture performed before or concomitantly to ceftaroline initiation in the case of empirical therapy; (ii) patients with MRSA-BSI, in line with the secondary objective of assessing response to treatment in this subgroup.

The onset of the infection in patients with MRSA-BSI was defined as the day when the first positive blood culture was drawn. A favorable response at the end of ceftaroline treatment in patients with MRSA-BSI was defined as complete or partial resolution of signs and symptoms of BSI.

### 2.2. Data Collection

The following demographic, clinical, and laboratory data was collected from the laboratory database and the patients’ medical records at the time of ceftaroline initiation: age in years; gender; Charlson comorbidity index [8]; previous solid organ transplantation; previous hematopoietic stem cell transplantation; previous colonization/infection by methicillin-susceptible *Staphylococcus aureus* (MSSA); previous colonization/infection by MRSA; previous therapy with ceftaroline; previous therapy with daptomycin; previous therapy with glycopeptides; previous therapy with linezolid; previous length of hospital stay in days; ward of stay; presence of central venous catheter (CVC) from at least 48 h; presence of urinary catheter from at least 48 h; use of mechanical ventilation from at least 48 h; presence of COVID-19 (defined by a positive real-time polymerase chain reaction for SARS-CoV-2 on at least one respiratory specimen); presence of neutropenia (defined as absolute neutrophil count <500/mm^3^); serum creatinine value; serum albumin value; Kidney Disease Improving Global Outcomes (KDIGO) stage of acute kidney injury [9]; Sequential Organ Failure Assessment (SOFA) score [10]; presence of septic shock (according to The Third International Consensus Definitions for Sepsis and Septic Shock (Sepsis-3) criteria [11]); type of ceftaroline therapy (empirical vs. targeted (i.e., after isolation of the causative agent), first-line vs. salvage therapy, on-label vs. off-label; monotherapy vs. combination therapy (defined as administration of ceftaroline with at least another anti-MRSA agent)); type of infection, according to the Centers for Disease Control and Prevention (CDC)/National Healthcare Safety Network (NHSN) surveillance definitions [12] (and with pneumonia being further divided into community-acquired pneumonia [CAP], hospital-acquired pneumonia [HAP] and ventilator-associated pneumonia [VAP] according to standard definitions [13]); type of clinical suspicion (e.g., sepsis) in the absence of data meeting CDC/NHSN criteria; results of cultures from specimens collected before/concomitant to ceftaroline initiation to pursue etiological diagnosis; genus and species of causative agent/s; and susceptibility to ceftaroline of MRSA isolates (the VITEK-2 automated system (bioMérieux, Marcy l’Etoile, France) was employed for the identification of isolates and for susceptibility testing).

In the subgroup of patients with MRSA-BSI, the following cross-sectional and longitudinal data was also collected: days elapsed from the onset of infection to initiation of ceftaroline; duration of ceftaroline therapy; presence of metastatic infection (defined as the presence of microbiological or radiographical evidence of *S. aureus* infection caused by hematogenous seeding); source control within 24 h from the onset of infection (CVC removal, abscess drainage, surgery); results of follow-up blood cultures performed at 72 h after ceftaroline initiation; clinical response at the end of ceftaroline treatment; mortality at the end of ceftaroline treatment; and mortality at 28 days after the onset of infection.

### 2.3. Sample Size Calculation and Statistical Analysis

By assuming normal distribution of estimated proportions for the primary descriptive, cross-sectional analysis (e.g., proportion of patients receiving ceftaroline for off-label indications/all patients receiving ceftaroline), a sample size of 200 patients would have guaranteed a maximum margin of error (confidence interval) of ± 7% with α = 0.05, and was ultimately considered as an acceptable compromise between feasibility and generalizability of study results.

The use of ceftaroline in the entire study population and in the subgroup of COVID-19 patients, as well as the rates of clinical response in patients with MRSA-BSI, were summarized with numbers and percentages for categorical variables, and with median and interquartile ranges for continuous variables. The 95% confidence interval (CI) was calculated for both proportions [14] and median values [15] estimates.

## 3. Results

The demographic and clinical characteristics of the 200 enrolled patients are presented in Table 1. Their median age was 66 years (interquartile range (IQR) 57–76), and 72% were males (144/200). As shown in the table, most of the patients had COVID-19 (83%, 165/200) and were hospitalized in medical wards (78%, 155/200).

**Figure 1:**
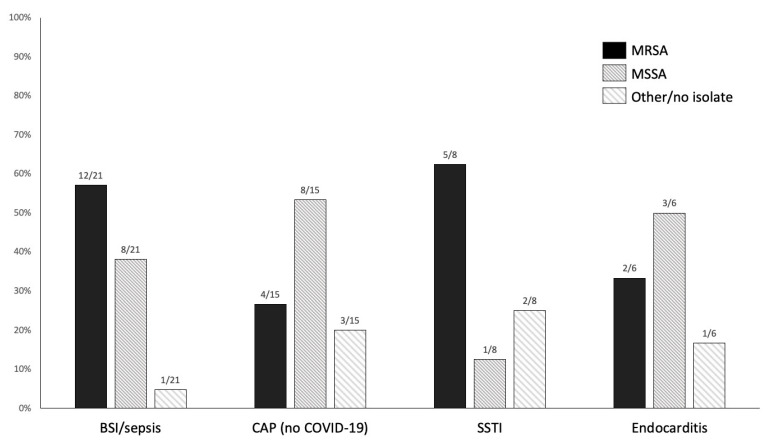
Distribution of MRSA and MSSA as etiological agents in patients without coronavirus disease 2019 (COVID-19) treated with ceftaroline. The figure includes etiological agents isolated both before and after initiation of ceftaroline therapy (i.e., isolates both from patients receiving targeted therapy and from patients receiving empirical ceftaroline with subsequent isolation of the causative agent). Indications are not mutually exclusive. BSI, bloodstream infection; CAP, community-acquired pneumonia; MRSA, methicillin-resistant *Staphylococcus aureus;* MSSA, methicillin-susceptible *Staphylococcus* aureus; SSTI, skin and soft tissue infection.

As shown in Table 2, there were two distinct modalities of ceftaroline prescriptions: (i) empirical therapy, the most frequent, that was mostly registered in patients with COVID-19 (165/179 empirical prescriptions, 92%); and (ii) targeted therapy, which was prescribed only in patients without COVID-19 (21/21 targeted prescriptions, 100%). Included patients with COVID-19 (i.e., those COVID-19 patients receiving empirical ceftaroline, whereas COVID-19 patients not receiving ceftaroline were not enrolled) had unilateral or bilateral consolidative pulmonary lesions at chest X-ray and/or computerized tomography and were given ceftaroline in the suspicion of community-acquired pneumonia (CAP), either as co-infection or superinfection to COVID-19. As reported in Table 3, etiological diagnosis of *S. pneumoniae* by urinary antigen was made in 1/140 tested COVID-19 patients. Respiratory specimens for culture were collected in only 13/165 COVID-19 patients (8%) and were positive in three of them (in two cases cultures yielded *Enterobacter aerogenes*, while *Pseudomonas aeruginosa* was isolated in the third case). In patients without COVID-19, targeted therapy was more frequent than empirical therapy, with MRSA being responsible for 63% (5/8), 57% (12/21), 33% (2/6), and 27% (4/15), of skin and soft tissue infections, bloodstream infections, endocarditis, and CAP, respectively (see Figure 1).

Among patients with MRSA-BSI, ceftaroline was used as first-line therapy and salvage therapy in 25% (3/12) and 75% (9/12) of cases, respectively, and as monotherapy or in combination with another anti-MRSA agent (in all cases with daptomycin) in 58% (7/12) and 42% (5/12) of patients, respectively (see Table 4). MRSA-BSI isolates were susceptible to ceftaroline (minimum inhibitory concentration range 0.25–1 mg/L). A favorable response at the end of ceftaroline therapy was registered in 67% (8/12) of patients, and 28-day mortality was 33% (4/12).

## 4. Discussion

The present cross-sectional study was conceived as an effort to depict the real-life use of ceftaroline within on-label and off-label indications. The concomitance of the COVID-19 pandemic allowed us to address an additional descriptive endpoint, that being the use of ceftaroline in COVID-19 patients with suspected bacterial CAP.

Most patients in our cohort were COVID-19 patients with pulmonary consolidative lesions receiving empirical ceftaroline. The use of antibiotics in COVID-19 patients has (and still is) much debated, with current epidemiological data depicting a low prevalence of bacterial infections and correctly pointing towards a more tailored use of antibiotics in COVID-19 patients with highly suspected/proven bacterial coinfection/superinfection [16,17,18]. In our opinion, the present study brings some additional points to this debate that are worth discussing. The first is that, while it is true that only in 4 out of 165 COVID-19 patients an etiological diagnosis was achieved (i.e., one positive urinary antigen for *Streptococcus pneumoniae* and three respiratory cultures yielding gram-negative rods), only 13 out of 165 COVID-19 patients underwent respiratory cultures, and this could have led to an important underestimation of the true prevalence of bacterial co-infections in our cohort. The second point, connected to the previous one, is that as many as 62%, 36% and 34% of respiratory cultures, blood cultures, and urine specimens for antigen testing, respectively, were collected after ceftaroline initiation, likely reducing their sensitivity for ceftaroline-susceptible bacteria. Finally, it is of note that, owing to the pressing need to provide rapid molecular testing results for SARS-CoV-2 in an overcrowded hospital during the peaks of the COVID-19 pandemic, the overworked laboratory was unable to perform additional molecular tests for other viruses and bacteria on respiratory specimens, further severely hampering the overall ability to identify true cases of bacterial CAP in COVID-19 patients. The overall picture is therefore dual: (i) on the one hand, the possible overlapped presentation of SARS-CoV-2 pneumonia (which may show consolidative pulmonary lesions independent of any possible bacterial co-infection/superinfection) and bacterial pneumonia may dictate toward the use of empirical antimicrobials in patients with severe disease, as was the case in our cohort; (ii) on the other hand, our results further remind us of the essential role of adequate laboratory testing for bacterial co-infection and superinfection in all COVID-19 patients with severe disease, possibly before initiation of empirical antibiotics. Only a comprehensive and timely approach to the diagnosis of bacterial infections may allow depiction of the true prevalence of bacterial CAP in COVID-19 patients, as well as allowing either targeted administration or rapid de-escalation of empirical antibiotics in specific cases, in line with the principles of personalized medicine.

Regarding the administration of ceftaroline in non-COVID-19 patients, despite the limitation of the small sample size, it is of note that most ceftaroline prescriptions were for off-label use, mostly for MRSA-BSI. Notably, ceftaroline was used for salvage therapy in 75% of cases, and its use was distributed in a similar way between monotherapy and combination therapy with daptomycin (58% vs. 42%, respectively). In our opinion, this highlights the persistent uncertainty in the literature, based on still inconclusive evidence, of the best approach (monotherapy vs. combination of daptomycin or vancomycin plus a beta-lactam) for the salvage therapy (or, in some cases, for first-line therapy) of MRSA-BSI. Indeed, while on the one hand the combination of vancomycin plus flucloxacillin was associated with reduced time to bacteremia resolution compared to vancomycin monotherapy in a RCT of 60 patients with MRSA-BSI, on the other hand another RCT comparing the combination of vancomycin or daptomycin plus an anti-staphylococcal beta-lactam (oxacillin, flucloxacillin, or cefazolin) vs. daptomycin/daptomycin monotherapy was terminated early due to safety concerns (increased rate of acute kidney injury in the combination arm) [19,20]. Concerning the specific addition of ceftaroline to daptomycin/vancomycin for the treatment of MRSA-BSI, in a recent retrospective study of 60 patients with MRSA-BSI, the combination of ceftaroline plus daptomycin was associated with lower odds of clinical failure (OR 0.23, with 95% CI 0.06–0.89) in multivariable analysis compared to the standard of care (vancomycin or daptomycin with or without the addition of trimethoprim/sulfamethoxazole, clindamycin, gentamicin, rifampin, or linezolid) [21]. Other observational studies have reported comparable/favorable success rates in patients with MRSA-BSI receiving ceftaroline in combination with daptomycin vs. daptomycin monotherapy [22,23,24,25,26]. While such a possible favorable effect is in line with the possible presence of the “seesaw effect” (i.e., improvement of β-lactam susceptibility in the presence of reduced daptomycin/vancomycin susceptibility) [27], it should be acknowledged that both in vitro and clinical observational results are hypothesis-generating at most, and dedicated, large RCTs remain ultimately necessary to disentangle the uncertainty about the use of ceftaroline either as a monotherapy or in combination for MRSA-BSI. In a small pilot RCT conducted in patients with MRSA-BSI, in-hospital mortality was 0% (0/17) and 26% (5/19) in patients treated with daptomycin plus ceftaroline vs. vancomycin or daptomycin monotherapy, respectively [28]. Overall, it is of note that, independent of ceftaroline use as a monotherapy or in combination, a high rate of favorable response (67%, 8/12) was observed in MRSA-BSI patients treated with ceftaroline in our cohort, and that there was no discontinuation of ceftaroline due to treatment-emergent adverse events.

In conclusion, we observed a dual major use of ceftaroline in our prospective study: (i) as empirical therapy for suspected bacterial CAP in COVID-19 patients; (ii) as targeted therapy for *S. aureus* infections (mostly MRSA-BSI) in non-COVID-19 patients. Improving respiratory diagnostic practices is essential to optimize the use of ceftaroline and other antibiotics in COVID-19 patients with highly suspected bacterial pulmonary coinfection or superinfection. The use of ceftaroline as salvage therapy for MRSA-BSI, either as a monotherapy or in combination with other anti-MRSA agents, shows promising rates of favorable response deserving further investigation through dedicated RCTs.

## Figures and Tables

**Table 1:** Demographic and clinical characteristics of patients treated with ceftaroline.

Variable	No. of Patients ^a^	%	95% CI
**Demographic Variables**			
Age in years, Median (IQR)	66 (57–76)		63–69
Male Gender	144/200	72	65–78
**Medical History**			
Charlson Score, Median (IQR)	3 (2–5)		3–3
Solid Organ Transplant	1/200	1	0–3
Hematopoietic Stem Cell Transplantation	1/200	1	0–3
Previous Isolation of MSSA	6/200	3	1–6
Previous Isolation of MRSA	5/200	3	1–6
Previous Therapy with Ceftaroline	2/200	1	0–4
Previous Therapy with Daptomycin	6/200	3	1–6
Previous Therapy with Glycopeptides	5/200	3	1–6
Previous Therapy with Linezolid	6/200	3	1–6
**Cross-sectional Variables ^b^**			
Length of Hospital Stay in Days, Median (IQR)	1 (1–3)		1–2
Ward of Staying			
ICU	34/200	17	12–23
Medical Ward	155/200	78	71–83
Surgical Ward	2/200	1	0–4
Emergency Department	9/200	5	2–8
Presence of CVC ^c^	13/200	7	4–11
Presence of Urinary Catheter ^c^	36/200	18	13–24
Mechanical Ventilation ^c^	8/200	4	2–8
COVID-19	165/200	83	77–87
Neutropenia (ANC < 500/mm^3^)	1/200	1	0–3
Serum Albumin in g/dl ^d^, Median (IQR)	3.0 (2.5–3.4)		2.9–3.1
Missing (serum Albumin not tested)	56/200		
Serum Creatinine in mg/dl ^d^, Median (IQR)	0.9 (0.8–1.2)		0.9–1.0
KDIGO Stage of AKI			
No AKI	170/200	85	79–89
Stage 1	16/200	8	5–13
Stage 2 Stage 3	8/200 6/200	4 3	2–8 1–6
SOFA Score, Median (IQR)	2 (2–4)		2–3
Septic Shock	54/200	27	21–34

AKI, acute kidney injury; ANC, absolute neutrophil count; BSI, bloodstream infection; CAP, community-acquired pneumonia; CI, confidence intervals; CVC, central venous catheter; ICU, intensive care unit; IQR, interquartile range; KDIGO, Kidney Disease: Improving Global Outcomes; MRSA, methicillin-resistant *Staphylococcus aureus*; MSSA, methicillin-susceptible *Staphylococcus aureus*; SOFA, sequential organ failure assessment. ^a^ Results are presented as No. of patients/Total of patients unless otherwise indicated; ^b^ At the time of ceftaroline initiation; ^c^ From at least 48 h; ^d^ Last measured value before ceftaroline initiation.

**Table 2:** Characteristics of ceftaroline therapies at the start of administration.

Variable	No. of Patients ^a^	%	95% CI
**Type of therapy**			
Empirical therapy	179/200	90	84–93
Targeted therapy ^b^	21/200	10	7–16
First-line therapy	103/200	52	44–59
Salvage therapy	97/200	48	41–56
On-label therapy	186/200	93	89–96
Off-label therapy	14/200	7	4–11
Monotherapy	193/200	97	93–98
Combination therapy ^c^	7/200	3	2–7
**Indications for empirical therapy ^d^**			
Sepsis	5/179	3	1–6
CAP in patients without COVID-19	7/179	4	2–8
CAP in patients with COVID-19	165/179	92	87–95
Skin and soft tissue infection	2/179	1	0–4
Endocarditis	1/179	1	0–3
Other ^e^	2/179	1	0–4
**Indications for targeted therapy ^d^**			
BSI	16/21	76	55–90
CAP in patients without COVID-19	8/21	38	20–60
CAP in patients with COVID-19	0/21	0	0–20
Skin and soft tissue infection	6/21	29	13–51
Endocarditis	5/21	24	10–46
Other ^f^	6/21	29	13–51

BSI, bloodstream infection; CAP, community acquired pneumonia; CI, confidence intervals; COVID-19, coronavirus disease 2019; HAP, hospital-acquired pneumonia; IQR, interquartile range; VAP, ventilator-associated pneumonia. ^a^ Results are presented as No. of patients/Total of patients unless otherwise indicated; ^b^ Post-identification of the causative agent; ^c^ With other agents with anti-MRSA activity: daptomycin (*n* = 7); ^d^ Not mutually exclusive; ^e^ HAP (*n* = 1), septic arthritis (*n* = 1), ^f^ HAP (*n* = 1), osteomyelitis (*n* = 1), pleural empyema (*n* = 1), VAP (*n* = 1), vertebral osteomyelitis (*n* = 2).

**Table 3:** Microbiological procedures in patients with COVID-19 and suspected bacterial CAP empirically treated with ceftaroline.

Variable	No. of Patients	%	95% CI
**Blood Cultures**			
Blood Cultures Collected	83/165	50	43–58
Collection before Ceftaroline Initiation	56/83	67	57–67
Positive Blood Cultures ^a^	1/83	1	0–6
**Respiratory Cultures**			
Respiratory Cultures Collected ^b^	13/165	8	4–13
Collection before Ceftaroline Initiation	5/13	38	17–66
Positive Respiratory Tract Cultures ^c^	3/13	23	7–52
**Urinary Antigen for *Streptococcus Pneumoniae***			
Urinary Antigen for *Streptococcus Pneumoniae* Collected	140/165	85	79–90
Collection before Ceftaroline Initiation	92/140	66	58–73
Positive Urinary Antigen for *Streptococcus Pneumoniae*	1/140	1	0–4

CAP, community acquired pneumonia; CI, confidence intervals; COVID-19, coronavirus disease 2019. ^a^ Only pathogens possibly responsible for CAP were considered (e.g., positive blood cultures for coagulase-negative staphylococci were excluded): *Escherichia coli* (*n* = 1); ^b^ Bronchoalveolar lavage fluid culture (*n* = 11), tracheal aspirate culture (*n* = 1), not specified (*n* = 1); ^c^ *Enterobacter aerogenes* (*n* = 2), *Pseudomonas aeruginosa* (*n* = 1)

**Table 4:** Clinical characteristics and outcomes in patients with MRSA-BSI treated with ceftaroline.

Variable	No. of Patients ^a^	%	95% CI
**Characteristics of Ceftaroline Therapy**			
Empirical Therapy ^b^	2/12	17	3–46
Targeted Therapy	10/12	83	54–97
First-Line Therapy	3/12	25	7–54
Salvage Therapy	9/12	75	46–93
Monotherapy	7/12	58	29–82
Combination Therapy ^c^	5/12	42	18–71
Time to Ceftaroline Initiation in Days, Median (IQR)	6 (2–10)		2–10
Duration of Ceftaroline Therapy in Survivors, Median (IQR)	13 (11–14)		5–16
**Type Of Infection**			
Isolated BSI	4/12	33	12–63
BSI With Metastatic Foci of Infection ^d^	8/12	67	37–88
**Early Source Control ^e^**			
Performed or Unnecessary	7/12	58	29–82
No ^f^	5/12	42	18–71
**Follow-Up Cultures At 72 H After Ceftaroline Initiation**			
Follow-Up Cultures Performed	9/12	75	46–93
Microbiological Success ^g^	6/9	67	32–90
**Clinical Outcomes**			
Favorable Response at the End of Ceftaroline Therapy	8/12	67	37–88
Mortality at the End of Ceftaroline Therapy	2/12	17	3–46
28-Day Mortality	4/12	33	12–63

BSI, bloodstream infection; CI, confidence intervals; IQR, interquartile range; MRSA, methicillin-resistant *Staphylococcus aureus*. ^a^ Results are presented as No. of patients/Total of patients unless otherwise indicated; ^b^ Etiological diagnosis made after ceftaroline initiation; ^c^ With other agents with anti-MRSA activity: daptomycin (*n* = 5); ^d^ Endocarditis (*n* = 3); pneumonia (*n* = 1), septic arthritis (*n* = 1), vertebral osteomyelitis (*n* = 1), pneumonia plus septic arthritis (*n* = 1), pneumonia plus vertebral osteomyelitis (*n* = 1); ^e^ Performed within 24 h from BSI onset (defined as the time when the first positive blood culture was drawn); ^f^ central venous catheter removed later than 24 h after BSI onset (*n* = 3), infective endocarditis deemed as inoperable by the cardiac surgeon (*n* = 2); ^g^ Defined as negative blood cultures at 72 h after ceftaroline initiation.

## Data Availability

The data presented in this study are available on reasonable request from the corresponding author.

## Supplementary Materials

The following are available online at https://www.mdpi.com/article/10.3390/antibiotics10070763/s1, protocol deviations.

## References

[1] European Medicines Agency Zinforo 600 mg Powder for Concentrate for Solution for Infusion: Summary of Product Characteristics. https://www.ema.europa.eu/en/documents/product-information/zinforo-epar-product-information_en.pdf.

[2] Bassetti M., Russo A., Cilloniz C., Giacobbe D.R., Vena A., Amaro R., Graziano E., Soriano A., Torres A. (2020). Ceftaroline for severe community-acquired pneumonia: A real-world two-centre experience in Italy and Spain. Int. J. Antimicrob. Agents.

[3] Burnett Y.J., Echevarria K., Traugott K.A. (2016). Ceftaroline as Salvage Monotherapy for Persistent MRSA Bacteremia. Ann. Pharmacother..

[4] Cortes-Penfield N., Oliver N.T., Hunter A., Rodriguez-Barradas M. (2018). Daptomycin and combination daptomycin-ceftaroline as salvage therapy for persistent methicillin-resistant Staphylococcus aureus bacteremia. Infect. Dis..

[5] Gritsenko D., Fedorenko M., Ruhe J.J., Altshuler J. (2017). Combination Therapy With Vancomycin and Ceftaroline for Refractory Methicillin-resistant Staphylococcus aureus Bacteremia: A Case Series. Clin. Ther..

[6] Shafiq I., Bulman Z.P., Spitznogle S.L., Osorio J.E., Reilly I.S., Lesse A.J., Parameswaran G.I., Mergenhagen K.A., Tsuji B.T. (2017). A combination of ceftaroline and daptomycin has synergistic and bactericidal activity in vitro against daptomycin nonsusceptible methicillin-resistant Staphylococcus aureus (MRSA). Infect. Dis..

[7] Zasowski E.J., Trinh T.D., Claeys K.C., Casapao A.M., Sabagha N., Lagnf A.M., Klinker K.P., Davis S.L., Rybak M.J. (2017). Multicenter Observational Study of Ceftaroline Fosamil for Methicillin-Resistant Staphylococcus aureus Bloodstream Infections. Antimicrob. Agents Chemother..

[8] Charlson M.E., Pompei P., Ales K.L., MacKenzie C.R. (1987). A new method of classifying prognostic comorbidity in longitudinal studies: Development and validation. J. Chronic Dis..

[9] Kellum J.A., Lameire N., Group K.A.G.W. (2013). Diagnosis, evaluation, and management of acute kidney injury: A KDIGO summary (Part 1). Crit. Care.

[10] Vincent J.L., Moreno R., Takala J., Willatts S., De Mendonça A., Bruining H., Reinhart C.K., Suter P., Thijs L.G. (1996). The SOFA (Sepsis-related Organ Failure Assessment) score to describe organ dysfunction/failure. On behalf of the Working Group on Sepsis-Related Problems of the European Society of Intensive Care Medicine. Intensive Care Med..

[11] Singer M., Deutschman C.S., Seymour C.W., Shankar-Hari M., Annane D., Bauer M., Bellomo R., Bernard G.R., Chiche J.D., Coopersmith C.M. (2016). The Third International Consensus Definitions for Sepsis and Septic Shock (Sepsis-3). JAMA.

[12] CDC/NHSN Surveillance Definitions for Specific Types of Infections. https://www.cdc.gov/nhsn/pdfs/pscmanual/17pscnosinfdef_current.pdf.

[13] (2014). Pneumonia: Diagnosis and Management of Community- and Hospital-Acquired Pneumonia in Adults.

[14] Blaker H. (2000). Confidence curves and improved exact confidence intervals for discrete distributions. Can. J. Stat..

[15] Pereira C.A., Polpo A. (2012). MedOr: Order of Medians Based on Confidence Statements. arXiv.

[16] Lansbury L., Lim B., Baskaran V., Lim W.S. (2020). Co-infections in people with COVID-19: A systematic review and meta-analysis. J. Infect..

[17] Rawson T.M., Moore L.S., Zhu N., Ranganathan N., Skolimowska K., Gilchrist M., Satta G., Cooke G., Holmes A. (2020). Bacterial and Fungal Coinfection in Individuals with Coronavirus: A Rapid Review To Support COVID-19 Antimicrobial Prescribing. Clin. Infect. Dis..

[18] Vena A., Giacobbe D.R., Di Biagio A., Mikulska M., Taramasso L., De Maria A., Ball L., Brunetti I., Loconte M., Patroniti N.A. (2020). Clinical characteristics, management and in-hospital mortality of patients with coronavirus disease 2019 in Genoa, Italy. Clin. Microbiol. Infect..

[19] Davis J.S., Sud A., O’Sullivan M.V., Robinson J.O., Ferguson P.E., Foo H., Van Hal S.J., Ralph A.P., Howden B.P., Binks P.M. (2016). Combination of Vancomycin and beta-Lactam Therapy for Methicillin-Resistant Staphylococcus aureus Bacteremia: A Pilot Multicenter Randomized Controlled Trial. Clin. Infect. Dis..

[20] Tong S.Y., Lye D.C., Yahav D., Sud A., Robinson J.O., Nelson J., Archuleta S., Roberts M.A., Cass A., Paterson D.L. (2020). Effect of Vancomycin or Daptomycin with vs. Without an Antistaphylococcal Beta-Lactam on Mortality, Bacteremia, Relapse, or Treatment Failure in Patients With MRSA Bacteremia: A Randomized Clinical Trial. JAMA.

[21] Johnson T.M., Molina K.C., Miller M.A., Kiser T.H., Huang M., Mueller S.W. (2021). Combination ceftaroline and daptomycin salvage therapy for complicated methicillin-resistant Staphylococcus aureus bacteraemia compared with standard of care. Int. J. Antimicrob. Agents.

[22] Ahmad O., Crawford T.N., Myint T. (2020). Comparing the Outcomes of Ceftaroline Plus Vancomycin or Daptomycin Combination Therapy Versus Monotherapy in Adults with Complicated and Prolonged Methicillin-Resistant Staphylococcus Aureus Bacteremia Initially Treated with Supplemental Ceftaroline. Infect. Dis. Ther..

[23] McCreary E.K., Kullar R., Geriak M., Zasowski E.J., Rizvi K., Schulz L.T., Ouellette K., Vasina L., Haddad F., Rybak M.J. (2020). Multicenter Cohort of Patients with Methicillin-Resistant Staphylococcus aureus Bacteremia Receiving Daptomycin Plus Ceftaroline Compared with Other MRSA Treatments. Open Forum Infectious Diseases.

[24] Hornak J.P., Anjum S., Reynoso D. (2019). Adjunctive ceftaroline in combination with daptomycin or vancomycin for complicated methicillin-resistant Staphylococcus aureus bacteremia after monotherapy failure. Ther. Adv. Infect. Dis..

[25] Morrisette T., Lagnf A.M., Alosaimy S., Rybak M.J. (2020). A comparison of daptomycin alone and in combination with ceftaroline fosamil for methicillin-resistant Staphylococcus aureus bacteremia complicated by septic pulmonary emboli. Eur. J. Clin. Microbiol. Infect. Dis..

[26] Sakoulas G., Moise P.A., Casapao A.M., Nonejuie P., Olson J., Okumura C.Y., Rybak M.J., Kullar R., Dhand A., Rose W.E. (2014). Antimicrobial salvage therapy for persistent staphylococcal bacteremia using daptomycin plus ceftaroline. Clin. Ther..

[27] Molina K.C., Morrisette T., Miller M.A., Huang V., Fish D.N. (2020). The Emerging Role of beta-Lactams in the Treatment of Methicillin-Resistant Staphylococcus aureus Bloodstream Infections. Antimicrob. Agents Chemother..

[28] Geriak M., Haddad F., Rizvi K., Rose W., Kullar R., LaPlante K., Yu M., Vasina L., Ouellette K., Zervos M. (2019). Clinical Data on Daptomycin plus Ceftaroline versus Standard of Care Monotherapy in the Treatment of Methicillin-Resistant Staphylococcus aureus Bacteremia. Antimicrob. Agents Chemother..

