# Supplementary material for: Use of Ceftaroline in Hospitalized Patients with and without COVID-19: A Descriptive Cross-Sectional Study
Source: Antibiotics (Basel). 2021 Jun 23;10(7):763. doi: 10.3390/antibiotics10070763 (PMC8300614; doi:10.3390/antibiotics10070763)
Supplement: Supplementary file 1 [file antibiotics-10-00763-s001.zip › antibiotics-1268982-supplementary.pdf]

antibiotics

MDPI

Supplementary Methods for

Article

Use of Ceftaroline in Hospitalized Patients with and without COVID-19: A Descriptive Cross-sectional Study

Daniele Roberto Giacobbe [1,2]*, Chiara Russo [1,2], Veronica Martini [3], Silvia Dettori [1,2], Federica Briano [1,2], Michele Mirabella [2], Federica Portunato [2], Chiara Dentone [2], Sara Mora [4], Mauro Giacomini [4], Marco Berruti [1,2] and Matteo Bassetti [1,2]

[1] Department of Health Sciences, University of Genoa, 16132 Genoa, Italy; chiara.russo16@icloud.com (C.R.); silvidetto@gmail.com (S.D.); federica.briano91@gmail.com (F.B.); marco.berruti1@gmail.com (M.B.); matteo.bassetti@unige.it (M.B.)
[2] Clinica Malattie Infettive, San Martino Policlinico Hospital—IRCCS, 16132 Genoa, Italy; Michelemirabella90@gmail.com (M.M.); fedu90@gmail.com (F.P.); chiara.dentone@hsanmartino.it (C.D.)
[3] School of Medicine, University of Genoa, 16132 Genoa, Italy; martini.vero@libero.it
[4] Department of Informatics Bioengineering, Robotics, and Systems Engineering (DIBRIS), University of Genoa, 16145 Genoa, Italy; sara.mora.1994@gmail.com (S.M.); Mauro.Giacomini@dibris.unige.it (M.G.);
* Correspondence: danieleroberto.giacobbe@unige.it

Protocol deviations

The following post-hoc deviations from the original protocol should be acknowledged:

The study was originally designed as a multicenter study (5 centers) based on local data of ceftaroline consumption. Owing to the increased burden of clinical duties consequent to the COVID-19 pandemic, the other 4 centers were eventually unable to complete ethics approval procedures and/or providing data. Nonetheless, data from the promoting center was ultimately sufficient to reach the predefined sample size of 200 patients

Among secondary analyses listed in the original protocol was the assessment of factors associated with use of ceftaroline in combination with other anti-MRSA (methicillin-resistant *Staphylococcus aureus*) agents; however, this analysis was eventually not conducted owing to the low number of patients receiving ceftaroline in combination with other anti-MRSA agents (see study results).
